# Bias in Discontinuous Elevational Transects for Tracking Species Range Shifts

**DOI:** 10.3390/plants14020283

**Published:** 2025-01-20

**Authors:** Shixuan Li, Jiannan Yao, Yang Lin, Siyu Wu, Zhongjie Yang, Chao Jin, Yuhan Zhang, Zhen Wang, Jinliang Liu, Guochun Shen, Mingjian Yu

**Affiliations:** 1 Zhejiang Tiantong Forest Ecosystem National Observation and Research Station, School of Ecological and Environmental Sciences, East China Normal University, Shanghai 200241, China; shixuanli1231@163.com (S.L.); 51253903002@stu.ecnu.edu.cn (J.Y.); 18289665799@163.com (S.W.); superking_jin@163.com (C.J.); 2 College of Life Sciences, Zhejiang University, Hangzhou 310058, China; linyang19990816@163.com (Y.L.); isamarezyh@163.com (Y.Z.); wangz158@163.com (Z.W.); fishmj@zju.edu.cn (M.Y.); 3 College of Life Sciences, Zhejiang Normal University, Jinhua 321004, China; 4 College of Life Sciences, China Jiliang University, Hangzhou 310018, China; 15949603508@163.com; 5 College of Life and Environmental Science, Wenzhou University, Wenzhou 325035, China; jinliang.liu@foxmail.com; 6 Shanghai Institute of Pollution Control and Ecological Security, 1515 North Zhongshan Road (No. 2), Shanghai 200092, China

**Keywords:** species migration, species distribution range, woody plant, altitudinal transect, estimation bias

## Abstract

Climate change is compelling species to seek refuge at higher elevations and latitudes. While researchers commonly study these migrations using discontinuous elevational transects, this methodology may introduce significant biases into our understanding of species movement. These potential biases could lead to flawed biodiversity conservation policies if left unexamined. To address this concern, we utilized species distribution data from a novel continuous elevational transect to evaluate the accuracy of discontinuous transect methods. Our analysis focused on how quadrat spacing and survey time intervals affect bias in estimating species range shifts. The results were striking: the widely used settings for discontinuous transects failed to detect 7.2% of species, inaccurately estimated shift distances for 78% of species, and produced an overall error rate of 86%. Wider quadrat spacing increased these error rates, while longer survey intervals generally reduced them. Moreover, discontinuous transects consistently underestimated species shift distances, with this underestimation becoming more pronounced over longer survey periods. Our pioneering assessment of bias in discontinuous elevational transects demonstrates that a 50 m quadrat spacing combined with a 60-year survey interval optimizes monitoring species range shifts for conservation planning. This baseline protocol could be further strengthened through supplementary, frequent surveys targeting high-elevation species—a strategic approach that maximizes accuracy while maintaining cost-effectiveness.

## 1. Introduction

Global warming is driving widespread shifts in species distribution ranges along elevational and latitudinal gradients [1,2,3]. While some studies documented downward migration in certain species [4], the predominant response to macroclimate warming across many species is an upward shift in elevation, especially among those adapted to warm conditions [3,5]. These shifts can lead to a variety of ecological consequences, including range contractions [6], local extinctions [7,8], and changes in community composition and species interactions [9,10]. Mountain ecosystems, often harboring unique species found nowhere else, are particularly vulnerable to climate change, especially at higher elevations [11]. These ecosystems also have sharp temperature and precipitation gradients [12,13]. Therefore, they allow for the observation of species range shifts over shorter distances along elevational gradients, making them ideal platforms for studying the response of species distribution to global warming [1,14].

Previous studies on species range shifts along mountain elevation gradients have primarily relied on discontinuous elevational transects. These transects, composed of multiple quadrats spaced at regular elevational intervals (e.g., 100 m) [15], offer several advantages, including ease of establishment, accessibility along trails or roads, and cost-effectiveness. Additionally, non-linear transect designs (L-, V-, or W-shaped) can further improve the capture of habitat heterogeneity along the elevational gradient [16,17]. However, the large elevation gaps between quadrats limit the detection of species restricted to these gaps or range shifts occurring within them [2,3,15]. Moreover, the discontinuous sampling design of quadrats can influence biodiversity estimation accuracy [18,19]. Consequently, monitoring species range shifts using discontinuous transects in mountain ecosystems may introduce biases of unknown magnitude. Some studies even suggested that the rapid and extensive nature of species range shifts may outpace our current monitoring ability [20], hindering our understanding of how species respond to climate change and the underlying mechanisms.

**Figure 1:**
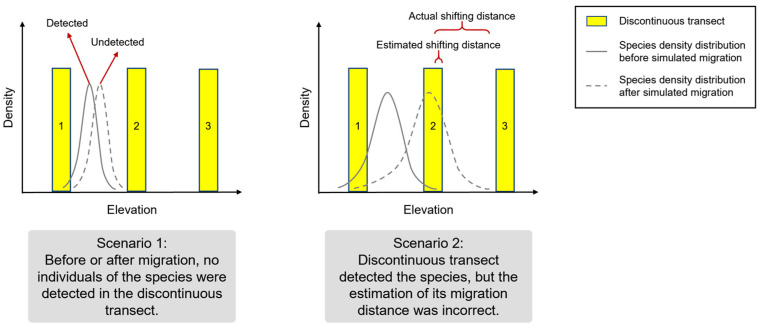
Two scenarios illustrating potential biases in estimating species range shifts using discontinuous elevational transects. The *x*-axis represents a continuous elevation gradient, and the *y*-axis represents the individual density of species along elevation. Yellow rectangles denote the quadrats of the discontinuous transect. The solid gray line shows the species’ initial distribution, while the dashed gray line represents the distribution after a simulated range shift.

We believe that biases in estimating species range shifts using discontinuous transects can primarily arise from two scenarios. Scenario 1: Undetected Species: A species’ entire distribution range may lie between two quadrats, leading to its undetected presence. As shown in the left panel of Figure 1, before the range shift, a small number of individuals were present in quadrat 1 (solid gray line). However, after the shift (dashed gray line), all individuals are distributed between quadrats 1 and 2, resulting in the species being missed by the discontinuous transect. Scenario 2: Underestimated Range Shift: Discontinuous transects can underestimate the magnitude of a species’ range shift. As depicted in the right panel of Figure 1, before the shift, the discontinuous transect correctly identifies the upper limit of the species’ distribution within quadrat 2. After the shift, while the actual upper limit approaches quadrat 3, the transect may still detect it only within quadrat 2 due to the absence of individuals in quadrat 3. This leads to a significant underestimation of the upward shift in the species’ range.

The magnitude of these two estimation biases is theoretically linked to the spatial spacing of quadrats in discontinuous elevational transects and the temporal frequency of surveys. When survey intervals are fixed, increasing the distance between quadrats may lead to a higher proportion of individuals being missed, potentially resulting in the undetected presence of species with narrow elevational ranges. Additionally, wider spacing can reduce the sensitivity of discontinuous transects to detect species range shifts. While increasing quadrat spacing can reduce survey effort, it can also compromise the accuracy of range shift estimates [19]. Conversely, if quadrat spacing remains constant but survey intervals are short, estimates of species range shifts might be significantly biased too. Over short time periods, species may migrate short distances, and survey error in tree elevational position can substantially influence range shift estimates. Furthermore, short-distance range shifts occurring between quadrats may be difficult to detect with discontinuous transects. To mitigate these factors, most studies employing discontinuous elevational transects collect species survey data over longer time spans (e.g., 50–100 years) [2,6,21].

Given the aforementioned potential for biases and theoretical drivers of bias, it is surprising that we lack a comprehensive understanding of how these biases manifest in discontinuous elevational transects and how they might be minimized through adjustments to quadrat spacing and survey intervals. Our study aims to bridge these gaps by quantitatively assessing these biases and their relationships with sampling design. Specifically, recognizing that species distribution patterns can influence bias estimation [22], we collected comprehensive species distribution datasets along a continuous elevation gradient (see Section 3.2). By combining these data with simulations of species migration, we addressed the following questions: First, does estimating species range shifts based on discontinuous transects introduce bias, and if so, to what extent? Second, how do quadrat spacing and survey intervals affect the degree of estimation bias? We hypothesize that larger quadrat spacing increases bias while longer survey intervals decrease it. Lastly, what are the optimal quadrat spacing and survey intervals for minimizing bias in species range shift estimation?

## 2. Results

### 2.1. Bias by Common Survey Method

**Figure 2:**
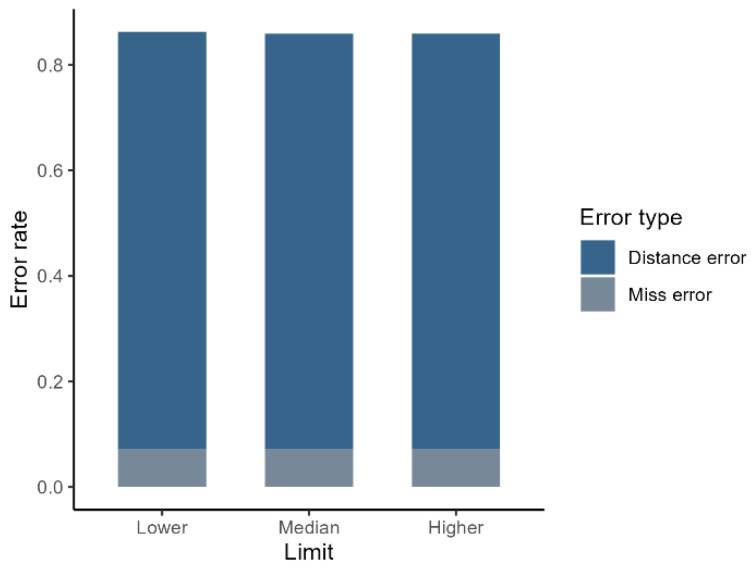
Error rates in estimating species distribution range shifts using discontinuous elevational transects with 100 m quadrat spacing and a 5-year survey interval. The *x*-axis represents the different boundaries of the species distribution range: “Lower” indicates the lower elevation limit, “Median” indicates the median elevation, and “Higher” indicates the higher elevation limit. The *y*-axis represents the error rate, with different colors representing different types of errors, as illustrated in Figure 1.

Discontinuous elevational transects with 100 m quadrat spacing and 5-year survey intervals miss 7.2% of species and significantly underestimate shift distances for 78–79% of detected species (relative deviation > 0.3). This results in an overall error rate of approximately 86% (Figure 2). Importantly, in this case, error rates are similar across the lower, median, and higher elevation limits of species distribution ranges (Figure 2).

### 2.2. Impact of Quadrat Spacing on Estimating Bias

**Figure 3:**
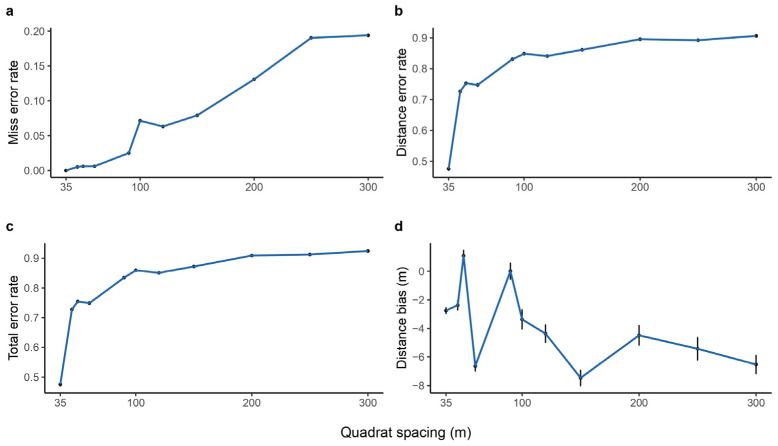
Error rates in estimating species median elevation shifts using discontinuous elevational transects under a survey interval of 5 years and various quadrat spacings. The *x*-axis represents the spacing of quadrats in discontinuous transects: (**a**) the error rate of missed species, (**b**) the error rate of shifting distance, (**c**) the total error rate, and (**d**) the distance bias along quadrat spacing with ±1.96 error bars.

As quadrat spacing increases (fixed 5-year interval), discontinuous transects become less effective in estimating median elevation shifts. Species omission rates rise, reaching ~20% at 300 m spacing (Figure 3). Concurrently, the error in estimating shift distances increases and then tends to level off, reaching ~90% at 300 m spacing (Figure 3b). This results in a total error rate of ~93% at 300 m spacing (Figure 3c). Furthermore, bias in shift distance estimates changes from overestimation at smaller spacings to underestimation at larger spacings (Figure 3d). Error rates for higher and lower elevation limits also increase with quadrat spacing, similar to the median elevation (see Supplementary Figures S1 and S2). However, underestimation is more pronounced for higher limits than lower limits (see Supplementary Figures S1d and S2d).

### 2.3. Impact of Survey Interval on Estimating Bias

**Figure 4:**
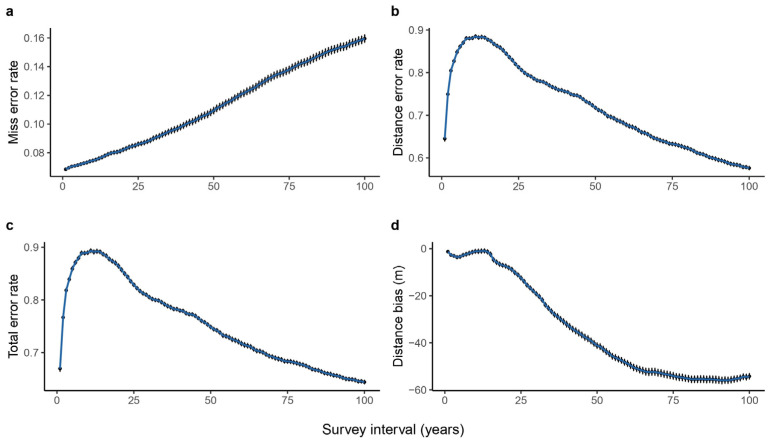
Error rates of estimating species median elevation shifting using discontinuous elevational transects under 100 m quadrat spacing and various survey intervals. The *x*-axis represents the time interval of transect surveys in discontinuous transects: (**a**) the error rate of missed species, (**b**) the error rate of shifting distance, (**c**) the total error rate, and (**d**) the distance bias along quadrat spacing with ± 1.96 error bars.

Increasing survey intervals (fixed 100 m spacing) generally reduces the effectiveness of discontinuous transects in estimating median elevation shifts. Species omission rates increase with longer survey intervals: annual surveys result in the fewest misses (6.9%), while 100-year intervals significantly increase the rate to 16% (Figure 4a). The error in estimating shift distances peaks at over 88% for approximately decadal surveys, decreasing for both shorter and longer intervals (Figure 4b). This results in a total error rate peak of 90% for around 10-year intervals (Figure 4c). Discontinuous transects consistently underestimate shift distances, with the degree of underestimation increasing to over 54 m for 80-year intervals (Figure 4d). Biases in estimating higher and lower elevation limit shifts also fluctuate with survey intervals. However, underestimation is more pronounced for higher limits, reaching 77% error at 100-year intervals, while lower limit underestimation is less severe (32% at 100 years) (see Supplementary Figures S3b and S4b). Notably, 13–32-year intervals can lead to a slight overestimation of lower limit shifts (see Supplementary Figure S4d).

### 2.4. Bias Under Combinations of Different Quadrat Spacings and Survey Intervals

**Figure 5:**
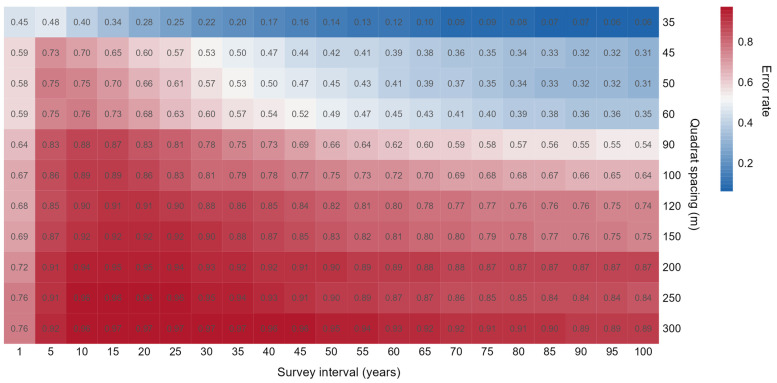
Heatmap of the total error rates in estimating species median elevation shifts using discontinuous elevational transects, varying with different combinations of quadrat spacings and survey intervals. The *y*-axis represents the spacing between quadrats, the *x*-axis represents the survey time interval, and the values within the cells represent the total error rate.

The aforementioned simulations separately assessed biases due to quadrat spacing and survey interval. To examine their combined impact, we assessed various combinations of both factors. Generally, smaller quadrat spacing and larger survey intervals lead to more accurate estimates of species distribution range shifts, except within the 1–10-year interval range, where shorter intervals can be less biased (Figure 5). Biases in estimating shifts in higher and lower elevation limits are similar to those for the median elevation, but higher limits generally exhibit greater bias (Figure 5; see Supplementary Figures S5 and S6). However, overall, significant biases persist in estimating species range shifts using discontinuous elevational transects.

## 3. Materials and Methods

We established a comprehensive set of continuous elevational gradient transects in the Baishanzu National Park, China, and conducted a thorough individual tree survey [23]. Utilizing this survey data, we simulated discontinuous elevational gradient transects by setting quadrats at equal elevation intervals. We then simulated individual tree deaths and births to mimic species range shifts, generating post-shift distribution patterns. By comparing these simulated shifts to those shifts estimated from discontinuous transects, we quantified the estimation bias from the two scenarios above. The detailed technical flowchart is shown in Supplementary Figure S7. All simulations and analyses were performed using R 4.3.0 (R Core Team, 2023).

### 3.1. Study Area

The Baishanzu National Park, a core component of the Qianjiangyuan-Baishanzu National Park, encompasses 50,351 ha in the southwest of Zhejiang Province, China (see Supplementary Figure S8). The park’s vegetation types follow a distinct elevational gradient, transitioning from evergreen broad-leaved forests at low elevations through evergreen and deciduous broad-leaved mixed forests to coniferous and broad-leaved mixed forests, coniferous forests, montane dwarf forests, shrublands, and grasslands at high elevation. This complete vertical zonation exemplifies the characteristic vegetation patterns of eastern subtropical mountain ecosystems [24].

The Baishanzu National Park experiences a subtropical maritime monsoon climate, receiving 1667.8–1804.6 mm annual precipitation with 1578.9–1699.8 h of yearly sunshine [24]. Despite its subtropical location, the park’s high elevation results in a cool average annual temperature of 18.2–18.3 °C [24]. The soil types vary with elevation, with zonal red soil predominating at low elevations and yellow soil at high elevations [25].

### 3.2. Vegetation Survey

We established a 3210 m continuous elevational transect in the Baishanzu National Park to facilitate long-term forest monitoring. The transect is 30 m wide and covers 9.63 ha, spanning elevations from 636 to 1928 m [24]. To optimize sampling efficiency, the transect is divided into three spatially discontinuous but continuous elevational segments, encompassing diverse ecosystems while avoiding human-disturbed and unstable areas (Figure S8). Lin et al. [24] found that elevation exerts the primary influence on species alpha and beta diversity along the transect, followed by slope, with aspect having a lesser impact.

To ensure long-term monitoring, the transect adheres to standard forest dynamic monitoring plot survey protocols [26,27]. The elevational gradient comprises 107 continuous 30 m × 30 m quadrats, each further divided into 36 smaller 5 m × 5 m subquadrats. Within each small quadrat, woody plants with a diameter at breast height (DBH) ≥ 1 cm were individually tagged, identified, mapped, and measured for DBH and height. Health assessments were also recorded. Pantropical families—notably Lauraceae, Theaceae, and Symplocaceae—dominate the transect, with north temperate families like Fagaceae and Adoxaceae forming a secondary component. At the species level, *Rhododendron simiarum*, *Rhododendron latoucheae*, and *Schima superba* exhibit the highest abundance [23]. The transect includes 69,914 free-standing trees: 260 woody plant species across 109 genera and 58 families. To enhance the reliability of our analysis, we excluded 68 species with fewer than 5 individuals, as their small populations and potential sensitivity to disturbances could introduce uncertainty into our error examination. The final dataset used for analyses comprised 192 woody plant species and 69,770 individuals.

### 3.3. Simulation of Discontinuous Elevational Transect and Species Range Shifting

**Figure 6:**
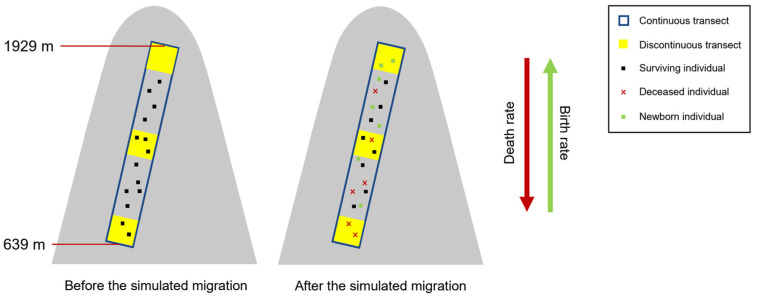
Schematic diagram of a discontinuous elevational transect and species range shift simulation. A continuous elevation transect (blue frame) is divided into evenly spaced quadrats (yellow squares) to simulate a discontinuous transect. Under a warming climate scenario, mortality rates decrease with increasing elevation while birth rates increase. Black dots represent individual trees. After the simulated migration, red crosses indicate dead individuals and green dots represent regenerated individuals. To simulate an upward elevation shift, a higher mortality rate is preferred at lower elevations, while a higher birth rate occurs at higher elevations.

To simulate discontinuous elevational transects, we established a series of quadrats along the 636–1928 m elevation gradient of the Baishanzu continuous elevational transect. These quadrats, each 30 m wide and 30 m in elevation, were spaced at regular intervals, ensuring coverage of the entire elevation range while mimicking the discontinuous nature of real-world transects (Figure 6). This approach allowed us to assess the impact of discontinuous sampling on estimating species range shifts. In real-world studies, quadrat placement may be influenced by factors that avoid the boundaries of syntaxa, potentially introducing bias into our results.

Our assessment of bias is based on simulated species range shifts, as our continuous elevational transect has been surveyed only once (2022–2023). Specifically, we used the observed elevational distribution of each species from the vegetation survey to establish initial species distributions along the elevational gradient in the simulation. Assuming a warming climate scenario, we modeled increased birth rates and decreased mortality rates with increasing elevation, thereby facilitating species migration towards higher elevation areas [28,29]. We set a base probability of 0.05 y^−1^ for both birth and mortality [30]. This parameterization, in conjunction with the elevation-dependent effects simulated below, allows for an average upward range shift of ~2.5 m∙y^−1^, which is consistent with observations from multiple studies [2,6]. To simulate an upward range shift, we employed a weighted probability approach. For each species, individuals were randomly selected for death or reproduction, with probabilities weighted by their relative elevation. Species that went extinct during the simulation were excluded from subsequent bias estimation. While actual species range shift patterns can be more complex, this study focuses on a simplified yet common upward migration scenario to assess the limitations of discontinuous transect methods [3].

### 3.4. Estimation of Species Distribution Range

To define a species’ distribution range, we determined the lower, median, and higher limits as the 0.05th, 0.5th, and 0.95th quantiles of its density distribution, respectively. This approach minimized the impact of outliers and provided a more accurate representation of the species’ distribution along the elevational gradient [6]. For each species and simulation, we rounded individual elevations to the nearest 0.5 m to account for potential measurement errors in field surveys. The actual distribution boundaries were calculated based on the species’ true distribution on the continuous transect. For the discontinuous transect, all individuals within a quadrat were combined to estimate the species’ distribution, and its boundaries were calculated accordingly. The true shift distance was determined by comparing the pre- and post-shift range distributions. Similarly, the estimated shift distance was calculated based on the pre- and post-shift estimated distributions from the discontinuous transect.

### 3.5. Calculation of Estimation Errors

If a species is undetected in any quadrat before or after the range shift, it is considered missed by the discontinuous transect, aligning with Scenario 1 in Figure 1. For detected species, we calculated the relative deviation of the shift distance as: Relative deviation = (Estimated shift distance − Actual shift distance)/Actual shift distance. If the actual shift distance was non-zero and the relative deviation exceeded 0.3, the discontinuous transect was considered to have an underestimated or overestimated shift, aligning with Scenario 2 in Figure 1. If the actual shift distance was zero but the estimated distance was non-zero, a distance error was considered. For each simulation, we assess both types of errors. The missed species error (Scenario 1) was calculated as the number of missed species divided by the total number of non-extinct species. The distance error (Scenario 2) was calculated as the number of species with distance errors divided by the total number of non-extinct and non-missed species. The total error rate was the sum of these two rates. After 999 repeated simulations, we calculated the average error rates for both scenarios.

Quadrat spacing in discontinuous elevational transects is typically around 100 m [2,15]. We initially used a 100 m quadrat spacing to assess the error rates of estimating species range shifts with a 5-year survey interval. We then explored how error rates varied with changes in quadrat spacing and survey intervals. For simplicity and consistency of results, we primarily presented error rates for the median elevation of species distributions in the main text. Additional results for lower and upper range limits were similar and were available in the Supplementary Materials.

## 4. Discussion

Our study clearly showed that commonly used discontinuous elevational transects significantly bias estimates of species range shifts. While we anticipated some bias, its extent, as shown in Figure 2, was unexpected. These biases mainly arise from gaps between quadrats in discontinuous transects. Species present within these gaps can lead to inaccurate estimates of range boundaries and shift distances. In severe cases, entire species may be missed. Our analysis shows that inaccurate shift distance estimates are more common than missed species, indicating that the primary bias in estimating range shifts from discontinuous transects stems from errors in shift distance estimation, rather than species omission. This finding also implies that if the primary objective of a study is to monitor species diversity along elevational gradients, the common design of discontinuous elevational transects may be sufficient, as only 7.2% of species were missed in our study.

Our study highlights the importance of avoiding excessively large quadrat spacing to prevent overlooking rare or narrowly distributed species [31]. Larger quadrat spacing can lead to significant biases in species distribution range estimates and, consequently, inaccurate shift distance calculations. As quadrat spacing increases, more species, especially those with narrow elevation ranges, are missed. This can lead to underestimates of species richness and diversity. Additionally, the accuracy of estimated shift distances declines. When quadrat spacing reaches a certain threshold, most species’ shift distances will be misestimated. Only a small subset of species, primarily those with very large or very small distribution ranges, may still be accurately assessed. These findings emphasize the need for careful consideration of quadrat spacing when designing surveys to monitor changes in species distribution ranges. Using discontinuous transects with large quadrat spacing can lead to substantial deviations from reality, potentially compromising the reliability of ecological assessments.

**Figure 7:**
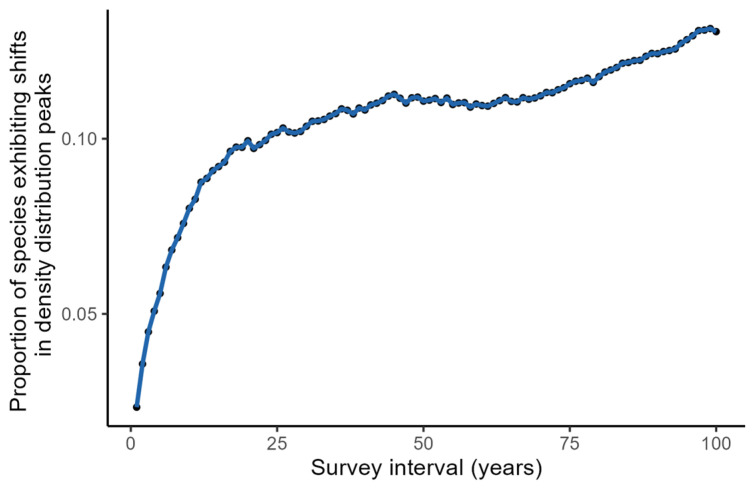
Proportion of species with density distribution peaks shifting into or out of small quadrats at a quadrat spacing of 100 m across varying survey intervals. The proportion was calculated as the sum of species initially present within quadrats but subsequently absent and species initially absent but subsequently present, divided by the total number of species consistently monitored throughout the study.

The increase in survey time interval leads to a surprising unimodal pattern in the error rate of estimating species shift distances using discontinuous transects. This complex pattern is driven by two competing factors: First, as species’ ranges shift, their density distribution peaks may move into or out of quadrats, leading to abrupt changes in estimated distribution ranges and increased error rates. Second, longer migration distances can increase the likelihood of detecting range shifts, as they reduce the impact of shifts occurring solely between quadrats and minimize survey error, potentially decreasing error rates. Specifically, for short survey intervals (e.g., 1 year), species migrate short distances, and their density distribution peaks rarely shift into or out of quadrats (Figure 7), resulting in relatively accurate estimates. As the survey interval increases, species migrate further, and the number of species experiencing shifts in density distribution peaks increases rapidly (Figure 7). Initially, the negative impact of shift-induced error outweighs the positive impact of migration-induced accuracy, leading to increased error rates, peaking around a 10-year interval (Figure 4b). However, as the survey interval continues to increase, the number of species experiencing shifts in density distribution peaks stabilizes. At this point, the positive impact of migration-induced accuracy becomes more dominant, reducing the error rate in estimating species range shifts using discontinuous transects.

Another drawback of long survey time intervals for discontinuous transects is that they can lead to greater underestimation of species shift distances (Figure 4d, see Supplementary Figures S3d and S4d). When the survey interval is short, the migration of individuals into or out of quadrats has a minimal impact on the overall community’s median elevation. However, as the survey interval increases, a significant number of high-elevation species migrate out of the highest-elevation quadrats. This results in an underestimation of these species’ range boundaries, with the degree of underestimation typically not exceeding the quadrat spacing (100 m in this case). Therefore, for conservation strategies that depend on accurate monitoring of species range shifts, we caution against employing extremely long survey intervals, such as the century-long periods used in some studies [32,33]. While more frequent ground surveys can significantly increase field costs, alternative cost-effective methods can complement traditional ground surveys. These include drone-based high-resolution remote sensing [34] and genetic marking [35], which are particularly effective for monitoring species with distinctive canopy colors or identifiable genetic markers.

Despite the value of discontinuous elevational transects as an ecological research platform [36], this study demonstrates that both quadrat spacing and survey time interval significantly influence the accuracy of estimated species range shifts. Therefore, careful consideration of these factors is crucial when employing discontinuous transects to study species migration. To balance accuracy and cost, a quadrat spacing of 50 m and a 60-year survey interval could be considered, resulting in an estimated error rate of around 40% for median elevation shifts. However, if the cost of establishing transects of such quadrat spacing is still unacceptable, then increasing the investment in time costs or appropriately sacrificing accuracy could be considered. For more accurate estimates within a shorter timeframe, reducing quadrat spacing is necessary. Alternatively, one can use continuous elevational transects to completely avoid these potential biases. While continuous transects offer greater precision for monitoring short-term disturbances, they require substantial investments in human and material resources.

We acknowledge that our study provided a preliminary assessment of biases in estimating species range shifts using discontinuous transects. Our simulations did not account for various factors, such as species-specific traits, land-use changes, and environmental disturbances (e.g., wildfires), which can influence migration patterns [2,37,38]. Moreover, the buffering effect of understory microclimates may interact with other factors, leading to a climatic lag or debt in forest communities, or some species may actually migrate to lower elevations [4,39,40]. Incorporating these factors could enhance the realism of our simulations and improve the accuracy of bias assessments. Furthermore, this study focused solely on the range shift of tree species, while recognizing that different biotypes may exhibit distinct responses to climate change [8,9]. Further research, incorporating other monitor planforms such as GLORIA [41], is necessary to fully understand these diverse responses. Additionally, the movement of species due to climate change is likely to modify floristic diversity, potentially requiring changes in syntaxa names as species compositions shift [42]. Therefore, our simulated range shifts may not fully capture real-world dynamics. Future re-surveys of the Baishanzu continuous elevational transect after five or ten years will provide valuable empirical data to refine our understanding of estimation biases and validate our simulation results.

## 5. Conclusions

In summary, this study revealed significant biases in estimating species range shifts using discontinuous elevational transects. Under common survey methods, a substantial portion of species were either missed (7.2%) or had their shift distances inaccurately estimated (78%), leading to a total error rate of 86%. These errors were exacerbated by larger quadrat spacing. While increasing the survey interval can mitigate some errors, it can also lead to severe underestimation of species shift distances. For a balance between accuracy and cost, a quadrat spacing of 50 m and a 60-year survey interval can yield an estimated error rate of around 40% for median elevation shifts.

## Data Availability

The data used for the simulation and analysis of this study are available from the corresponding author, G.S., upon reasonable request.

## Supplementary Materials

The following supporting information can be downloaded at: https://www.mdpi.com/article/10.3390/plants14020283/s1, Figure S1: Error rates in estimating species higher elevation limit shifts using discontinuous elevational transects under a survey interval of 5 years and various quadrat spacings; Figure S2: Error rates in estimating species lower elevation limit shifts using discontinuous elevational transects under a survey interval of 5 years and various quadrat spacings; Figure S3: Error rates of estimating species higher elevation limit shifting using discontinuous elevational transects under 100 m quadrat spacing and various survey intervals; Figure S4: Error rates of estimating species lower elevation limit shifting using discontinuous elevational transects under 100 m quadrat spacing and various survey intervals; Figure S5: Heatmap of the total error rates in estimating species higher elevation limit shifts using discontinuous elevational transects, varying with different combinations of quadrat spacings and survey intervals; Figure S6: Heatmap of the total error rates in estimating species lower elevation limit shifts using discontinuous elevational transects, varying with different combinations of quadrat spacings and survey intervals; Figure S7: Research method flowchart; Figure S8: Schematic diagram of study area and continuous elevational transect.
