# Supplementary material for: Bias in Discontinuous Elevational Transects for Tracking Species Range Shifts
Source: Plants (Basel). 2025 Jan 20;14(2):283. doi: 10.3390/plants14020283 (PMC11768634; doi:10.3390/plants14020283)
Supplement: Supplementary file 1 [file plants-14-00283-s001.zip › plants-3355841-supplementary.pdf]

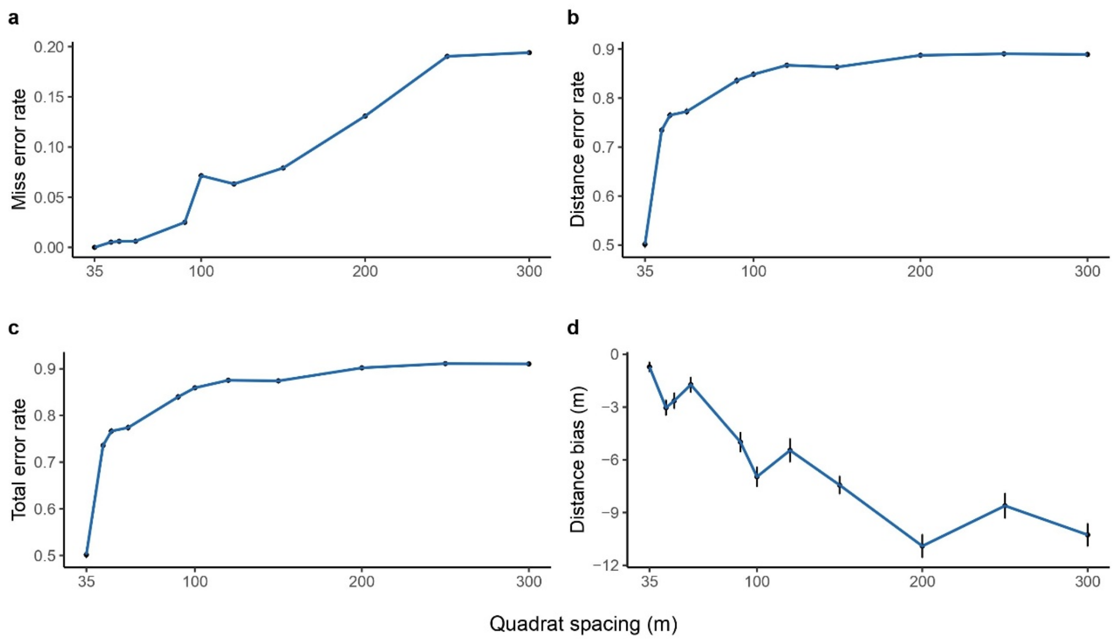

Figure S1. Error rates in estimating species higher elevation limit shifts using discontinuous elevational transects under a survey interval of 5 years and various quadrat spacings. The *x*-axis represents the spacing of quadrats in discontinuous transects: (**a**) the error rate of missed species, (**b**) the error rate of shifting distance, (**c**) the total error rate, and (**d**) the distance bias along quadrat spacing with ±1.96 error bars.

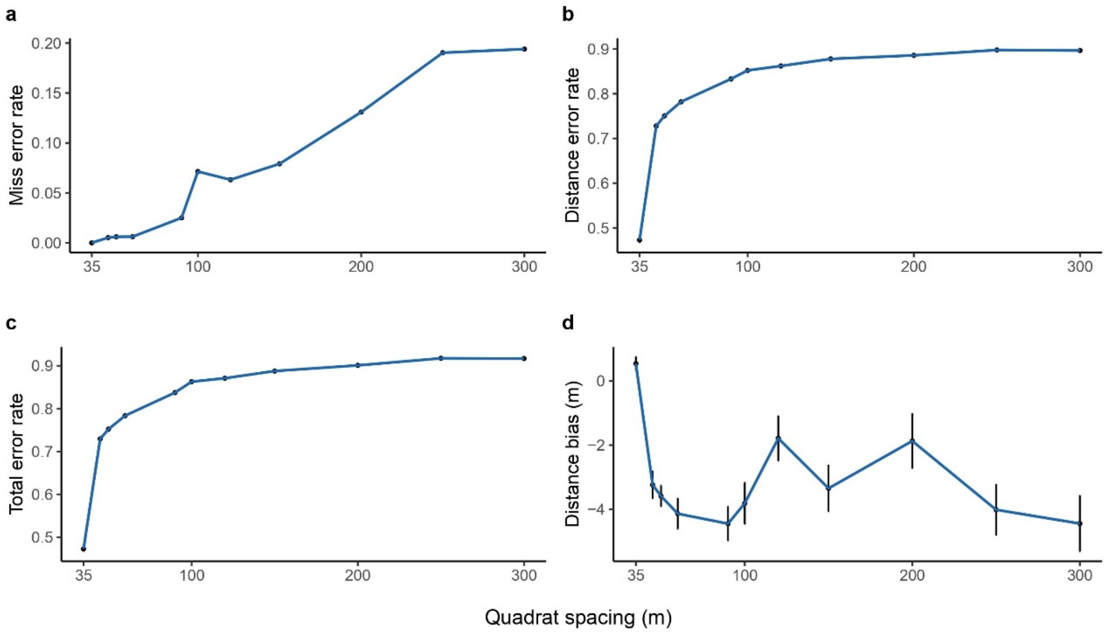

Figure S2. Error rates in estimating species lower elevation limit shifts using discontinuous elevational transects under a survey interval of 5 years and various quadrat spacings. The *x*-axis represents the spacing of quadrats in discontinuous transects: (**a**) the error rate of missed species, (**b**) the error rate of shifting distance, (**c**) the total error rate, and (**d**) the distance bias along quadrat spacing with ±1.96 error bars.

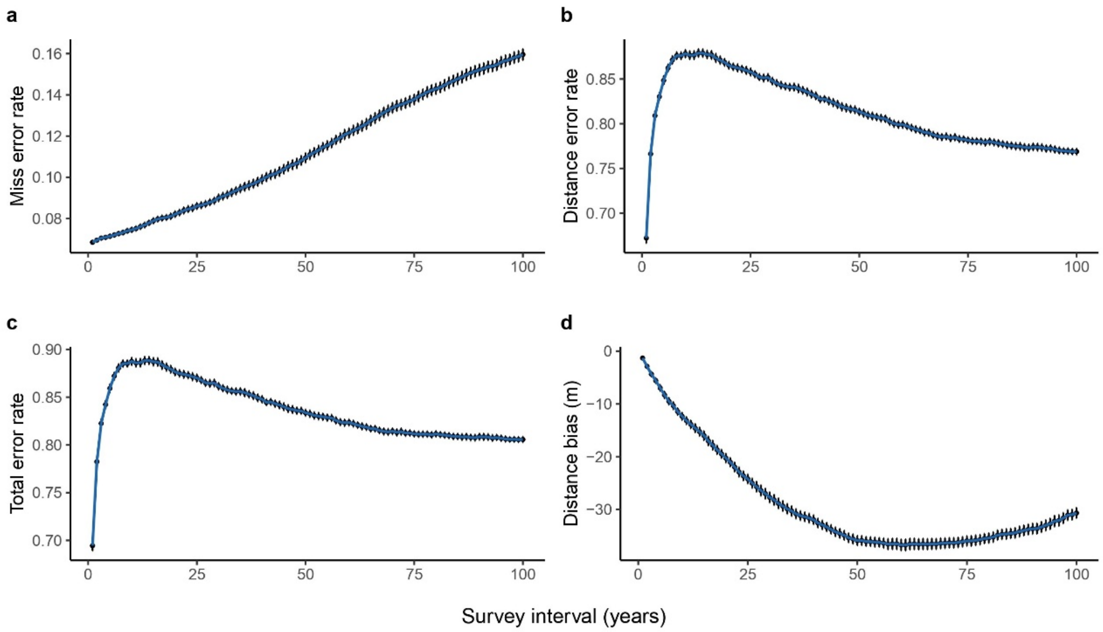

Figure S3. Error rates of estimating species higher elevation limit shifting using discontinuous elevational transects under 100 m quadrat spacing and various survey intervals. The *x*-axis represents the time interval of transect surveys in discontinuous transects: (**a**) the error rate of missed species, (**b**) the error rate of shifting distance, (**c**) the total error rate, and (**d**) the distance bias along quadrat spacing with ± 1.96 error bars.

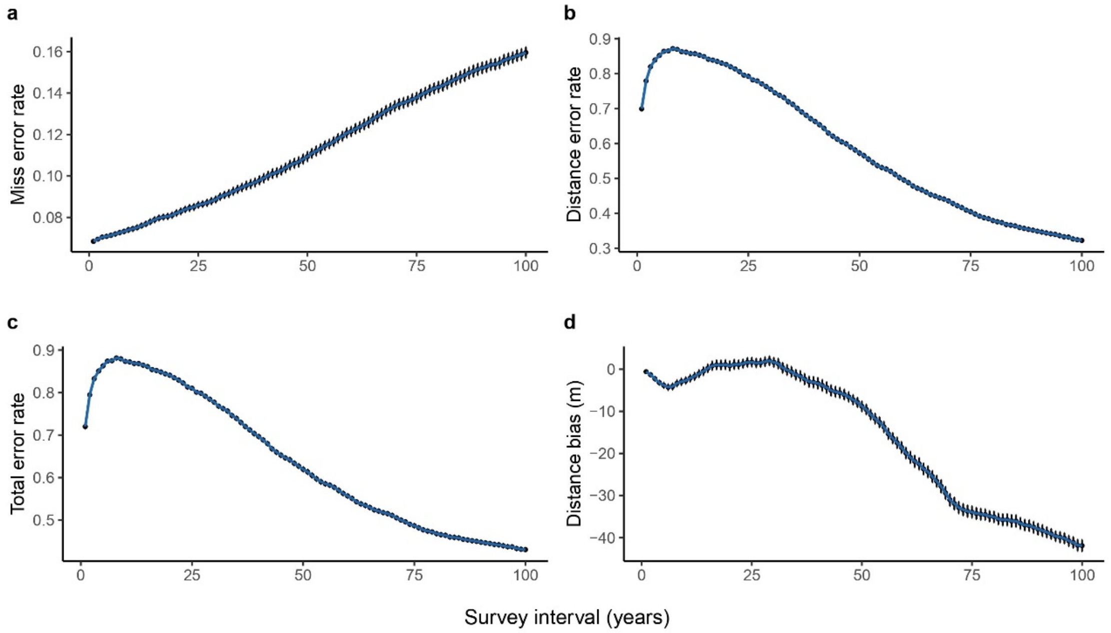

Figure S4. Error rates of estimating species lower elevation limit shifting using discontinuous elevational transects under 100 m quadrat spacing and various survey intervals. The *x*-axis represents the time interval of transect surveys in discontinuous transects: (**a**) the error rate of missed species, (**b**) the error rate of shifting distance, (**c**) the total error rate, and (**d**) the distance bias along quadrat spacing with ± 1.96 error bars.

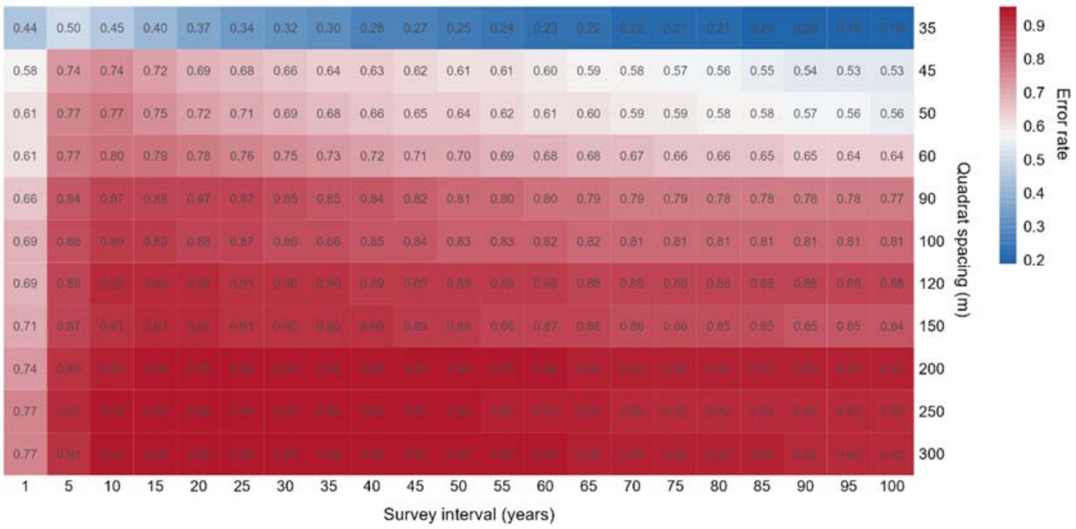

Figure S5. Heatmap of the total error rates in estimating species higher elevation limit shifts using discontinuous elevational transects, varying with different combinations of quadrat spacings and survey intervals. The *y*-axis represents the spacing between quadrats, the *x*-axis represents the survey time interval, and the values within the cells represent the total error rate.

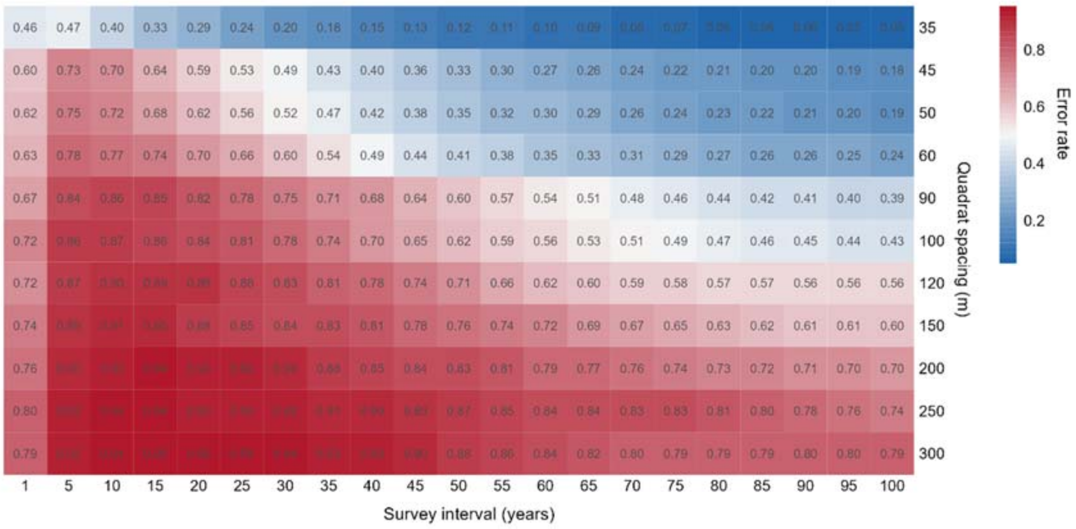

Figure S6. Heatmap of the total error rates in estimating species lower elevation limit shifts using discontinuous elevational transects, varying with different combinations of quadrat spacings and survey intervals. The *y*-axis represents the spacing between quadrats, the *x*-axis represents the survey time interval, and the values within the cells represent the total error rate.

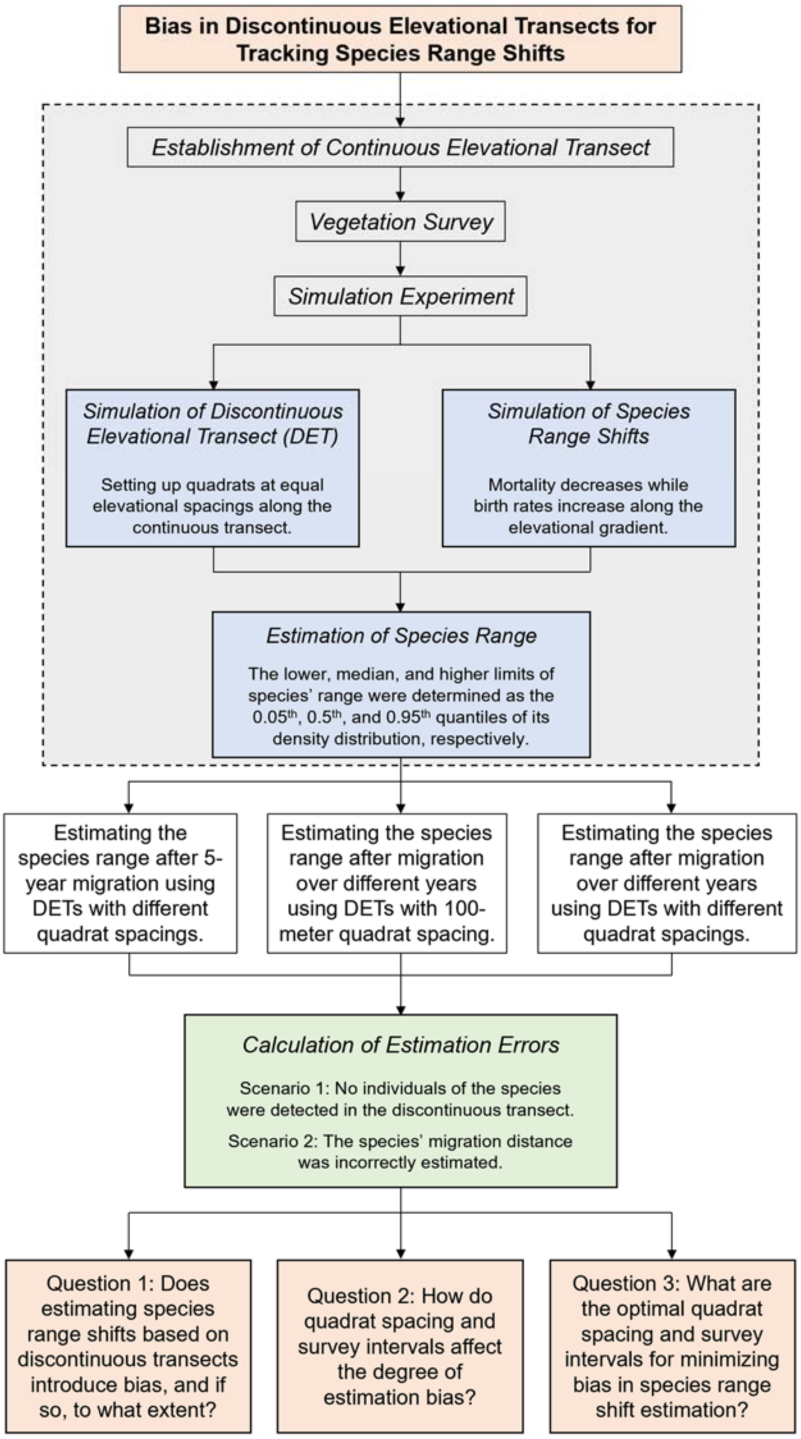

Figure S7. Research method flowchart.

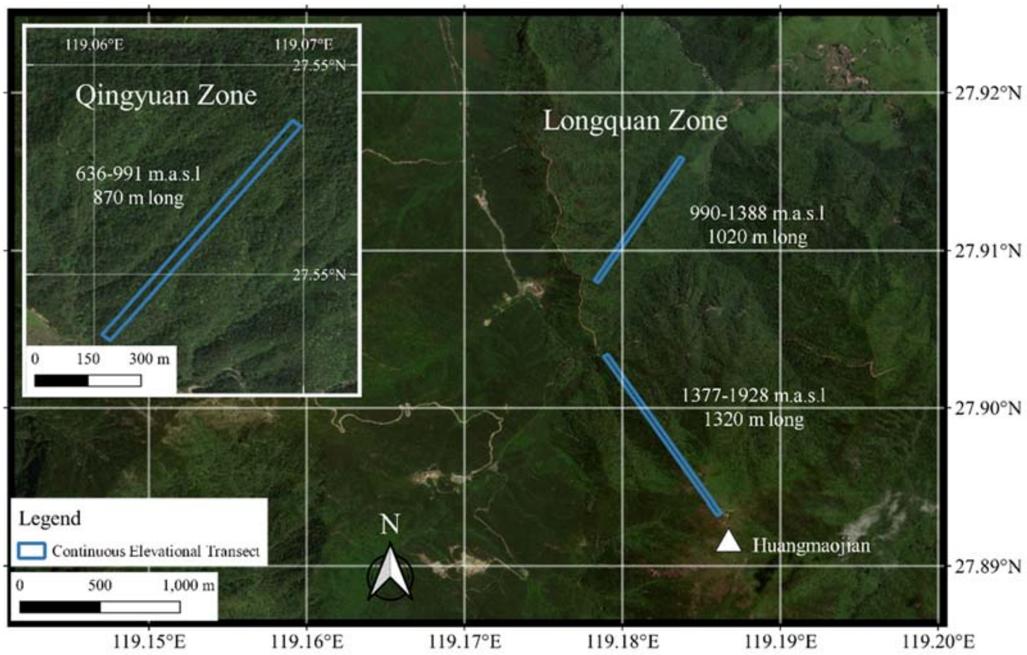

Figure S8. Schematic diagram of study area and continuous elevational transect. In the figure, "m.a.s.l" stands for "meter above sea level."
